# Development of organic fertilizers from food market waste and urban gardening by composting in Ecuador

**DOI:** 10.1371/journal.pone.0181621

**Published:** 2017-07-20

**Authors:** J. Jara-Samaniego, M. D. Pérez-Murcia, M. A. Bustamante, C. Paredes, A. Pérez-Espinosa, I. Gavilanes-Terán, M. López, F. C. Marhuenda-Egea, H. Brito, R. Moral

**Affiliations:** 1 Facultad de Ciencias, Escuela Superior Politécnica de Chimborazo, Riobamba, Chimborazo, Ecuador; 2 Dept. of Agrochemistry and Environment, Miguel Hernandez University, Orihuela, Alicante, Spain; 3 Facultad de Salud Pública, Escuela Superior Politécnica de Chimborazo, Riobamba, Chimborazo, Ecuador; 4 Dept. of Agri-Food Engineering and Biotechnology, Universitat Politècnica de Catalunya, Castelldefels, Spain; 5 Dept. of Biochemistry and Agrochemistry, Alicante University, San Vicente, Alicante, Spain; University of Notre Dame, UNITED STATES

## Abstract

Currently, the management of urban waste streams in developing countries is not optimized yet, and in many cases these wastes are disposed untreated in open dumps. This fact causes serious environmental and health problems due to the presence of contaminants and pathogens. Frequently, the use of specific low-cost strategies reduces the total amount of wastes. These strategies are mainly associated to the identification, separate collection and composting of specific organic waste streams, such as vegetable and fruit refuses from food markets and urban gardening activities. Concretely, in the Chimborazo Region (Ecuador), more than 80% of municipal solid waste is dumped into environment due to the lack of an efficient waste management strategy. Therefore, the aim of this study was to develop a demonstration project at field scale in this region to evaluate the feasibility of implanting the composting technology not only for the management of the organic waste fluxes from food market and gardening activities to be scaled-up in other developing regions, but also to obtain an end-product with a commercial value as organic fertilizer. Three co-composting mixtures were prepared using market wastes mixed with pruning of trees and ornamental palms as bulking agents. Two piles were created using different proportions of market waste and prunings of trees and ornamental palms: pile 1 (50:33:17) with a C/N ratio 25; pile 2: (60:30:10) with C/N ratio 24 and pile 3 (75:0:25) with C/N ratio 33), prepared with market waste and prunings of ornamental palm. Throughout the process, the temperature of the mixtures was monitored and organic matter evolution was determined using thermogravimetric and chemical techniques. Additionally, physico-chemical, chemical and agronomic parameters were determined to evaluate compost quality. The results obtained indicated that all the piles showed a suitable development of the composting process, with a significant organic matter decomposition, reached in a shorter period of time in pile 3. At the end of the process, all the composts showed absence of phytotoxicity and suitable agronomic properties for their use as organic fertilizers. This reflects the viability of the proposed alternative to be scaled-up in developing areas, not only to manage and recycle urban waste fluxes, but also to obtain organic fertilizers, including added value in economic terms related to nutrient contents.

## Introduction

The increasing generation of food waste and its management constitute a major concern. In the EU, around 100 million tonnes of food, mainly coming from homes but also from food markets, are wasted annually. This amount is expected to rise to over 120 million tonnes by 2020 if the situation does not change [1]. In developing countries the situation is even worst; the main food waste streams come from food market activities/areas, where it is usual to have a high production and a non-efficient waste management. In addition, these food waste streams are usually incorporated into the total municipal solid waste production. Bernache [2] reported a contribution of 9% of food market waste in the total municipal solid waste production in Guadalajara city (Mexico). During 2009, in the studied area (Chimborazo Region, Ecuador), only 15% of urban waste, which includes food market waste, was deposited in landfills and 85% was dumped into the environment, with about 14% of total municipal solid waste recycled by official and unofficial procedures [3]. This fact shows that although the legislation concerning environmental management is very recent in Ecuador, it is not very strict, being inapplicable in most cases. On the other hand, different authors have reported that the on site separate collection or “source separation” of the organic fraction of food market waste and municipal solid waste improves the characteristics of the end-products obtained and reduces the operating costs of the management treatment considered [4, 5, 6]. However, currently, in the Riobamba city, as in other areas of Ecuador, there is not selective collection of municipal solid waste. According to the municipal authorities, each vendor of the markets and free trade must deposit waste in suitable containers and then transfer them to larger deposits, which are transported to the open dump at the end of the day. These actions achieve greater cleanliness and order in the municipal markets, but not a suitable and optimized management of the wastes generated.

In the biggest market of the city of Riobamba (capital of the Chimborazo Region), as in other regions of Ecuador, different products that include vegetables, cereals, tubers, fruits, meat, dairy and foods in general are sold, mainly at whole scale. In this market, 6 tonnes of waste are daily generated, 95% (5.7 tonnes) being organic waste, which are currently disposed without any treatment into the open dump of the city (Data from the Departamento de Desechos Sólidos en Riobamba). The wastes from food market, principally constituted by vegetable waste, usually contain high levels of organic matter (OM), moisture, and nutrients [7]. In a previous study, Jara-Samaniego et al. [8] reported average values of 77.3% OM, and 2.5–0.7–3% N-P_2_O_5_-K_2_O nutrient values in food market wastes from the studied area (Chimborazo Region, Ecuador). In addition, a study in six markets in Morelia (Mexico) concluded that biodegradable compounds in food market waste were around 93% [9]. On the other hand, the generation of urban gardening waste is also increasing all over the world, and includes all the vegetable wastes generated during the operations of pruning-cutting and biomass removal. Boldrin and Christensen [10] reported for Denmark an increase of this type of organic wastes from 67 kg dry weight/person year in 1994 to 143 in 2006. This fact is not only due to the increase in the number of recreation areas, but also due to a better waste collection and data recording [11]. This residual biomass frequently includes a mixture from woody, caespitose, shrub and palm species with increasing recalcitrance to biodegradation. The huge amount in volume of this type of wastes, mainly due to its nature and density, the lack of pre-treatment and specific treatment facilities usually conclude in controlled disposal with significant losses of resources in terms of energy, nutrient and organic matter [12]. In many cases, uncontrolled open dumping and burning of residues are used as cheap solid waste management technique [13, 14]. This situation produces direct and indirect greenhouse gas emissions and other impacts in the environment and the human health. In Ecuador, the remains of shrubs and trees that are pruned regularly to avoid problems with electrical wiring and to maintain the urban parks and gardens are usually not treated and directly disposed in dumps.

Therefore, the management of these waste fluxes must be quick and efficient due to their high degradability, which makes unsuitable their disposal in municipal landfills. The disposal in landfills or dumpsites of these organic wastes, especially vegetable market waste, together with municipal solid wastes, generates different undesirable effects and negative impacts in the environment (bad odours, leachate production and greenhouse gas emission into the atmosphere, etc.) that affect the health of the population of the area of influence [13]. However, the high concentrations of organic matter and nutrient contents of food market and urban gardening wastes favour their recycling by composting, which allows to manage and recycle these materials, obtaining end-products (compost) for agricultural purposes. Composting is defined as the biological decomposition of organic matter under controlled aerobic conditions to form a stable, humus-like end product [15, 16]. Among the major waste management strategies developed, composting is gaining interest for organic waste disposal as a treatment with more economic and environmental profits, leading to a stabilized end product [13]. On the one hand, from the economic point of view, compost can be distributed in a wide variety of markets, especially when the demand for fertilizer is strongly correlated with high agricultural commodity prices [17]. As an example, Zhang et al. [18] demonstrated the role of composted green waste as a substitute for peat in growing media in state nurseries in Beijing (China). On the other hand, at an environmental level, composting not only avoids landfill for waste disposal, mitigating the formation of leachate and gases [19], but also implies the obtaining of a finished product that can enrich the soil and aid with water conservation [20]. However, although food waste and/or green waste composting may constitute a growing solution to solid waste management, many problems remain: the location of the composting facility, the suitable financial support for the installation and maintenance of the composting facility and/or the necessary technical knowledge, being the latter aspects especially problematic in developing countries, such as Ecuador. In this sense, composting is a technology with an easier implementation and lesser costs in relation to other technological options. Therefore, in countries with similar conditions to those as Ecuador, such as the lack of low-cost technological options for an efficient management of waste streams, it may be a win-win option for the management of clean organic waste streams, such as food market and urban pruning wastes, especially in specific agricultural uses of the composts obtained. However, despite composting can constitute a viable alternative method to manage this type of waste streams, not enough information is currently available on the composting of food market wastes and pruning waste in developing countries, and of the added value of the composts obtained.

Therefore, the main aim of this study were twofold: i) to evaluate and develop different co-composting strategies based on the use of food market wastes and pruning waste from urban gardening activities (including trees and palm species); ii) to obtain high quality composts under the conditions of the studied region, including an economical assessment of the NPK value content in these organic fertilizers.

## Materials and methods

### Experimental design

Three co-composting strategies were developed using food market wastes and wastes from urban gardening activities including tree pruning and palm pruning. All the food market wastes (MW) were collected in the San Pedro de Riobamba market (known as Mercado Mayorista), which is located in the south of the city of Riobamba (Chimborazo Region, Ecuador, -1°41'08.8"S; -78°37'58.3"W). The wastes were collected directly from vendors, with the authorisation of the Gobierno Autónomo Descentralizado Municipal de Riobamba, and were fresh vegetables remains: onion, artichoke, broccoli, spinach, cabbage, lettuce, garlic, basil, carrot, beet, cabbage, cauliflower, celery, radish, pepper, bean, corn, bean, pea, potato, and fruits such as apple, strawberry, blackberry, orange, banana, tomato, pineapple, mango, peach, melon, watermelon, lemon, avocado, grape, papaya, kiwi, plum, fig, lime. The improper materials, such as plastic, glass and paper were manually separated. Pruning wastes, tree prunings (TP) and palm prunings (PP) were collected separately in order to use them as bulking agents in the co-composting process. TP contained leaves and young branches of ornamental trees and shrubs dry (*Acacia* L., *Ficus* L. and *Populus* L.) of the avenues and parks of the city. PP came from ornamental palms (*Phoenix canariensis*). MW, TP and PP were homogenised and crushed using a crusher to obtain a particle size between 1 and 4 cm (Table 1).

**Table 1:** Main characteristics of the raw materials used (dry matter basis).

Parameter	MW	TP	PP	*F*-Anova
pH	7.85a	6.29a	5.56a	ns
EC (mS cm^-1^)	6.13b	2.62a	3.94ab	***
Organic matter (%)	84.5b	94.3c	65.9a	***
Corg (%)	45.9b	51.3c	35.8a	***
Nt (%)	1.81b	2.14c	0.86a	***
Ratio Corg/Nt	25.4a	23.9a	41.6b	***
R1 index	0.39c	0.20a	0.28b	***
P (%)	0.31a	0.15a	0.15a	**
WSPol (mg kg^-1^)	1710a	7794b	1749a	***
K (%)	2.21b	0.52a	0.74a	***
Ca (%)	1.16a	1.67b	1.62b	**
Mg (%)	0.25a	0.42b	0.39b	**
Fe (mg kg^-1^)	1063b	289b	4401c	***
Mn (mg kg^-1^)	52.5b	26.8a	66.2b	**
Cu (mg kg^-1^)	9.5a	6.4a	28.1b	***
Zn (mg kg^-1^)	29b	14a	53c	***

MW: food market waste; TP: tree prunings; PP: palm prunings; EC: electrical conductivity; Corg: total organic carbon; Nt: total nitrogen; R1 index: value that expresses the thermolability; WSPol: water-soluble polyphenols.

**, ***: Significant at P < 0.01, 0.001, respectively,

ns: Not significant. Mean values in rows followed by the same letter do not differ significantly at P < 0.05 (Tukey-b test).

The composting mixtures were mixed in the following proportions, on a fresh weight basis (dry weight basis in parenthesis):

Pile 1 (P1): 50% MW+33% TP+17% PP (16:57:27), C/N 26.5

Pile 2 (P2): 60% MW+30% TP+10% PP (22:59:19), C/N 25.8

Pile 3 (P3): 75% MW + 25% PP (33:67), C/N 32.9

The study was carried out at the open composting facility of the Environmental Theme Park Ricpamba (Riobamba-Ecuador). The mixtures (about 1000 kg each) were managed as trapezoidal windrows (about 1.8 x 3 x 1.5 m (length x width x height) and were mechanically turned when the temperature dropped to less than 40°C (8 turnings for P1 and P2; 4 turnings for P3), in order to homogenise and aerate the mixtures. The bio-oxidative phase of composting was considered finished when during 10 consecutive days after a whirl the difference between the pile temperature and the ambient temperature was ≤ 10°C. Then, composts were left to mature in static conditions over a period of one month, approximately. Throughout the composting process, temperature was monitored daily and the humidity of each pile was kept at optimal level for microbial metabolism (40–60%), by adding the necessary water. EXI was calculated as the summation of the daily value obtained by subtracting the ambient temperature from the temperature value in the composting pile during the bio-oxidative phase of composting, and expressed as cumulated °C. Composite samples were collected throughout the composting process, by taking, mixing and homogenizing ten subsamples from the whole profile of each mixture (from the top to bottom). All the samples were air-dried and ground to 0.5 mm prior to analysis.

### Analytical methods

In the initial materials and in the composting samples, pH and electrical conductivity (EC) were determined in the 1:10 (weight/volume) water soluble extract. The moisture content was analysed at 105°C for 24h. Organic matter (OM) was assessed by determining the loss-on ignition at 430°C for 24 h. Total organic carbon (Corg) was calculated according to the following equation C = OM/1.84 and total nitrogen (Nt) was determined by the Dumas method. After HNO_3_/HClO_4_ digestion, phosphorus (P) was assessed colorimetrically as molybdovanadate phosphoric acid, and potassium (K) was determined by flame photometry (Jenway PFP7 Flame Photometer, Jenway Ltd., Felsted, UK). The germination Index (GI) was calculated using seeds of radish (*Raphanus sativus* L.) [21]. Cation exchange capacity (CEC) was determined with BaCl_2_-triethanolamine according to the method described by [22]. Water-soluble polyphenols (WSPol) were determined by the modified Folin-Ciocalteu method in a 1:20 (w/v) water extract, according to Bustamante et al. [22].All the analyses were performed in triplicate.

The losses of OM were calculated using the initial (X_1_) and final (X_2_) ash contents, with the Eq (1) [23]:

$$OM\;loss\;(\%)=100\;-\;100\;\frac{\left[X_{1}\;\left(100\;-\;X_{2}\right)\right]}{\left[X_{2}\;\left(100\;-\;X_{1}\right)\right]}$$ (1)

For the thermogravimetric analysis (TG), samples were air-dried, ground in an agate mill and sieved through a 0.125 mm mesh, and milled again with an agate mortar. Thermal analyses were performed with a TGA/SDTA851e/LF/1600 instrument (Mettler Toledo, Barcelona, Spain) and Pfeiffer Vacuum (Thermostar GSD301T) mass spectrometer that enables the recording of thermograms and mass spectra of combustion gases simultaneously. All samples were combusted with a mixing stream of oxygen/He (20/80%), a gas flow 100 ml min^-1^ within a temperature range from 25 to 1000°C, a heating rate 10°C min^-1^, a sample weight about 5 mg, Al_2_O_3_ pan, and self-controlled calibration. All the assays were carried out in triplicate. The R1 index was calculated considering the overall loss of mass due to the loss of aliphatic materials (at 420 to 550°C) and carbohydrate molecules (at 200 to 420°C) during the combustion process.

### Economic value of compost nutrients

The economic value of the nutrients included in the composts was calculated considering the price of diammonium phosphate (DAP), standard size, bulk, spot, free on board (f.o.b.) US Gulf; potassium chloride (muriate of potash), standard grade, spot, f.o.b. Vancouver and Urea; Black Sea, bulk, spot, f.o.b. Black Sea (primarily Yuzhnyy) beginning July 1991; for 1985–91 (June) f.o.b. Eastern Europe, provided by the World Bank. Based on these materials, the value of 100 kg of each respective compound (N, P_2_O_5_ and K_2_O) was determined. Then, the economic value of the nutrients present in the composts was assigned, considering a content of 25% of fresh matter in all the composts.

### Statistical analysis

The characteristics of the initial materials were reported as the means of three replicates and were analysed using a one-way ANOVA design. To compare the differences, the Tukey-b test was used (P < 0.05). The normality and homogeneity of the variances were checked using the Shapiro-Wilk and Levene test, respectively, before ANOVA. The statistical analyses were conducted using the SPSS 20.0 software package.

OM losses during composting were fitted to a first-order kinetic function (Eq 2) by the Marquardt—Levenberg algorithm [24], a non-linear least-square technique, using the Sigmaplot 11.0 computer programme (Systat Software Inc. San Jose, California, USA):

$$\text{OM loss (\%)}=\text{A(1}-{\text{e}}^{\text{kt}}\text{);}$$ (2)

where A is the maximum degradation of OM (%), k the rate constant (d^-1^) and t the composting time (d). The RMS and F-values were calculated to compare the goodness of the curve fitting.

## Results and discussion

### Temperature evolution of the composting piles

**Fig 1:**
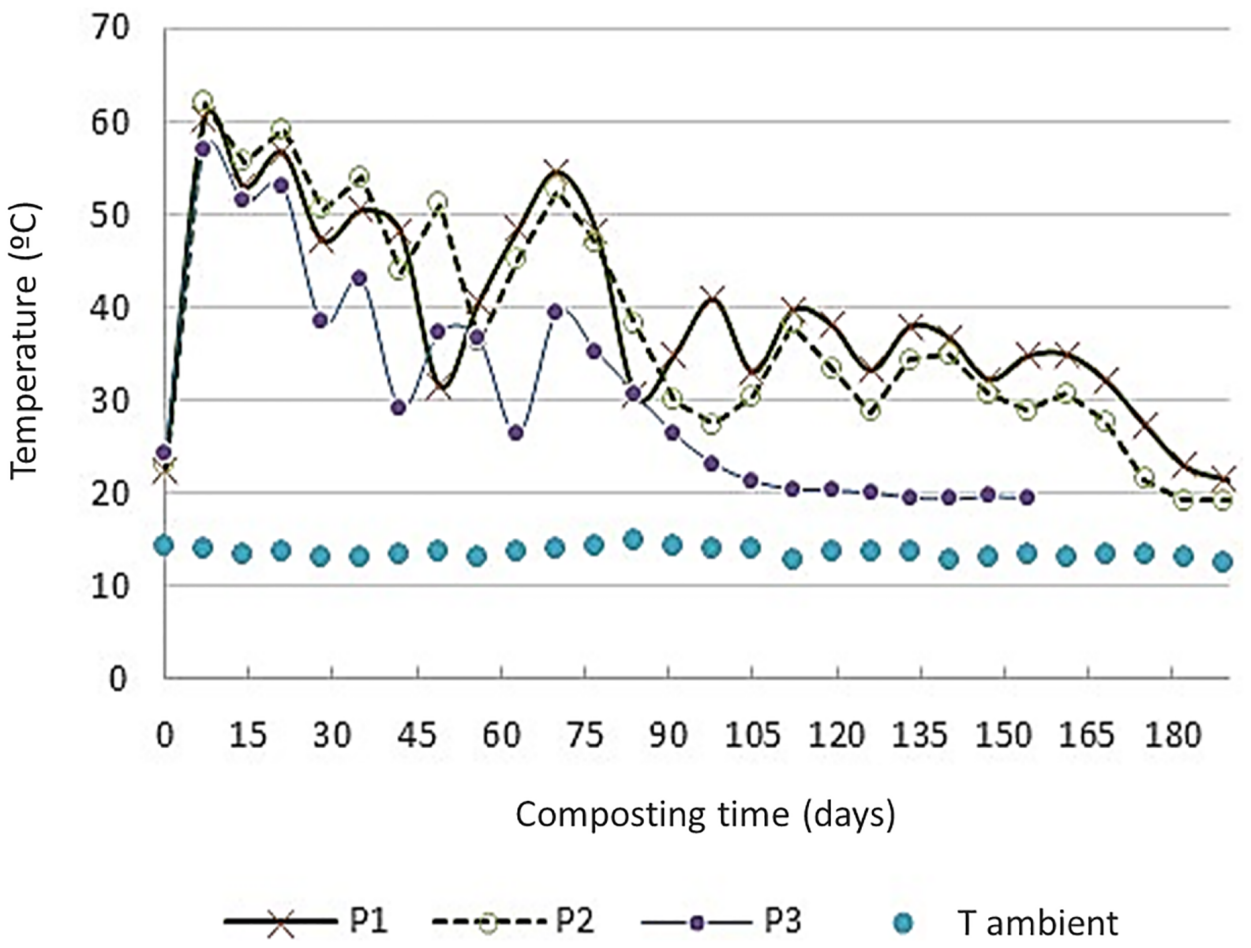
Temperature profiles of the composting piles. P1: 50% MW + 33% TP + 17% PP; P2: 60% MW + 30% TP + 10% PP; P3: 75% MW + 25% PP. MW: food market waste; TP: tree prunings; PP: palm prunings.

In all the mixtures, the temperature values showed a rapid increase during the first days of composting due to the microbial proliferation, reaching the highest temperatures in the first week (60.3°C, 62°C and 57.1°C, respectively for P1, P2 and P3) (Fig 1). Other authors have also reported this quick rise in the temperature values during composting of vegetable wastes [25, 26, 27]. In order to compare the increase of the temperature values in the three piles, the Exothermic Index (EXI) was used [28]. The end of the bio-oxidative phase was established when during 10 consecutive days after a whirl the difference between the pile temperature and the ambient temperature was ≤ 10°C. According to this criterion, the duration of the bio-oxidative phase was of 189, 182 and 105 days for P1, P2 and P3, respectively. The sequence for the values of EXI and of the ratio EXI/bio-oxidative period (days) were P1 (4995; 26.4) > P2 (4670; 25.7) > P3 (2457; 23.4). Thermal profiles of the piles seemed to be negatively affected by the proportion of MW in the composting mixture, EXI = -155.65 MW (% dry weight) + 7724.5 (n = 3, R^2^ = 0.9445). This can be attributed to the higher content of easily-degradable organic compounds provided by TP or PP compared to MW. In piles P1 and P2, the temperature exceeded 55°C for more than two weeks, which ensured the maximum pathogen reduction according to the European guidelines on compost sanitation [29]. P3 did not reach these requirements. However, the number of the days in thermophilic conditions, 63, 70 and 28 respectively ensured the correct evolution and stabilization of the organic matter.

### Organic matter losses

**Fig 2:**
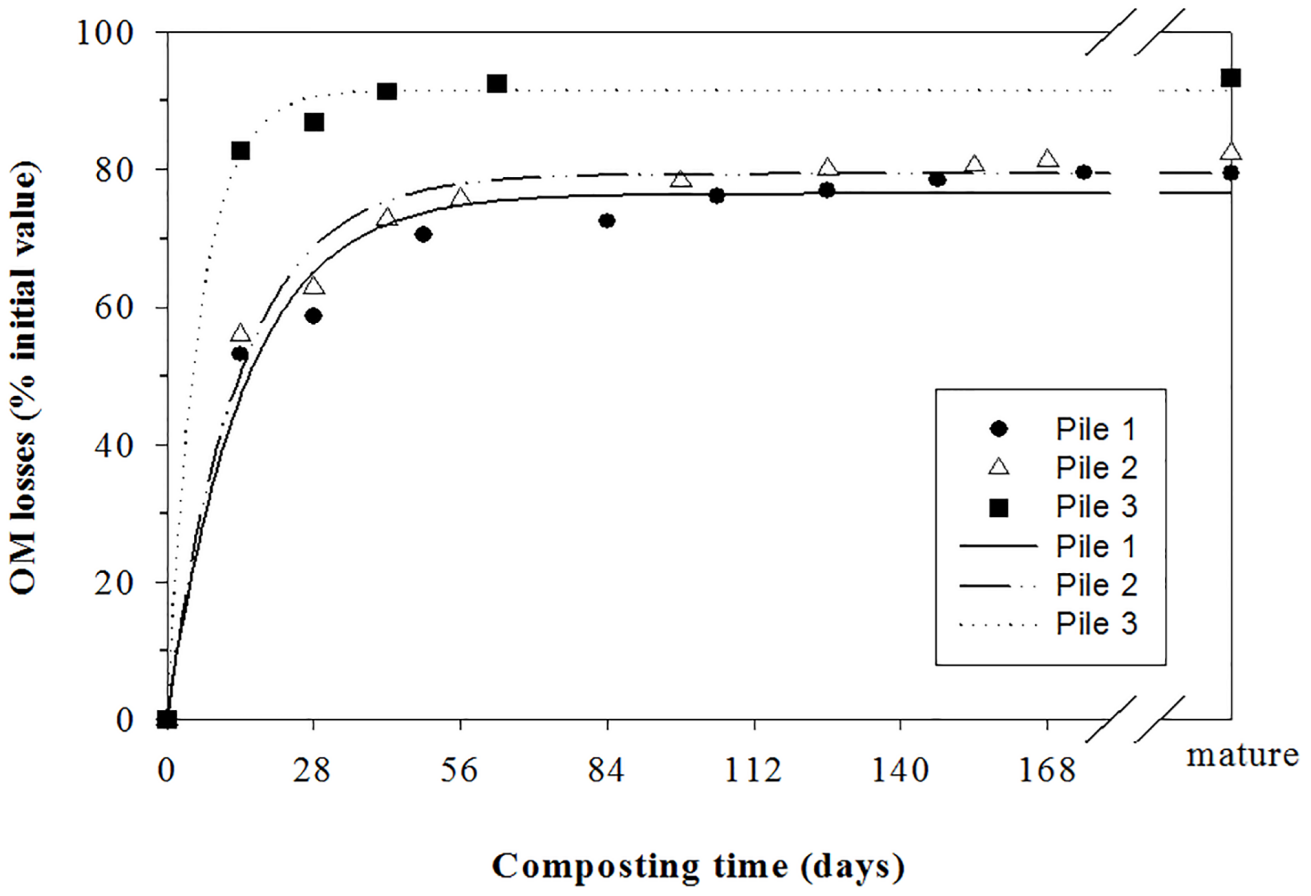
Organic matter losses during composting of piles. Lines represent curve-fitting. P1: 50% MW + 33% TP + 17% PP; P2: 60% MW + 30% TP + 10% PP; P3: 75% MW + 25% PP. MW: food market waste; TP: tree prunings; PP: palm prunings.

The initial concentrations of OM in the piles were 85.0% for P1, 84.9% for P2 and 95.7% for P3. The higher contents observed in P3 may be due to greater proportion of MW in this mixture; since this waste showed the higher concentrations of OM, together with TP (Table 1). The OM values decreased during the composting process, from the initial values previously commented to values of 53.8%, 49.6% and 59.8% for P1, P2 and P3, respectively, indicating the OM degradation [22]. OM losses were mainly found into the first month of composting (Fig 2), corresponding to the maximum microbial activity, reflected in the highest temperature values. This evolution was also reported by Bustamante et al. [22] and Gavilanes-Terán et al. [27] in experiments of composting of winery-distillery wastes and horticultural wastes, respectively. During the maturation phase, OM concentrations practically did not change, indicating the stability of the material after the bio-oxidative phase [22].

OM degradation profile during composting followed a first-order kinetic equation in all the piles. Curve fitting of the experimental data gave the following parameter values (standard deviation in brackets):

Pile 1:=76.5 (1.6), k =0.0678 (0.0082), RMS =0.9733, F =328.53***, SEE =3.99

Pile 2:=79.4 (1.4), k =0.0716 (0.0073), RMS =0.98134, F =473.62***, SEE =3.43

Pile 3:=91.5 (1.1), k =0.1613 (0.0177), RMS =0.9968, F =1541.16**, SEE =2.09

The highest values of A were observed for P3, related to the higher proportion of MW and PP. Similar behaviour were found for P1 and P2, according to the similar respective thermal profiles. Gavilanes-Terán et al. [27] reported similar A values (76–84%) during the co-composting of laying hen manure and sawdust mixed with broccoli and tomato waste. However, the k constant were significantly higher than the values found in other experiments of composting of vegetable wastes [22, 27, 30], indicating the higher biodegradability of these wastes. This could be a good indicator for the preselection of co-composting scenarios for composting plants focused on the most efficient OM reduction. In this sense, Jurado et al. [28] also reported for green waste composting an intense enzymatic activity during the first 2–3 weeks of composting (bio-oxidative phase), because of the availability of easily decomposable organic compounds.

### Evolution of the thermolability and compost quality parameters

Thermogravimetric analyses (TG) were carried out in the starting materials and in the composting samples to evaluate the chemical changes in the organic matter during the composting process. Thermolability is expressed by means of R1 index, defined as the ratio between the loss of mass in the range 420–550°C (due to aliphatic materials) in relation to the loss of mass in the range 200–420°C (due to carbohydrate molecules) during the combustion process [28]. Therefore, the R1 index allows to compare the most recalcitrant components with the most labile fraction.

As it has been commented, the first range of 200–420°C corresponds to the combustion of carbohydrates such as cellulose and lignocellulosic substances [26, 28, 31], which are the main components of the plant material. The thermolability values observed in the starting materials showed consistent results to validate this assumption, since the thermolability decreased in the sequence MW>PP>TP (Table 1). Probably, MW coming from markets in Ecuador includes lipid compounds, waxes and other recalcitrant components related to plant composition, especially for tropical species.

**Fig 3:**
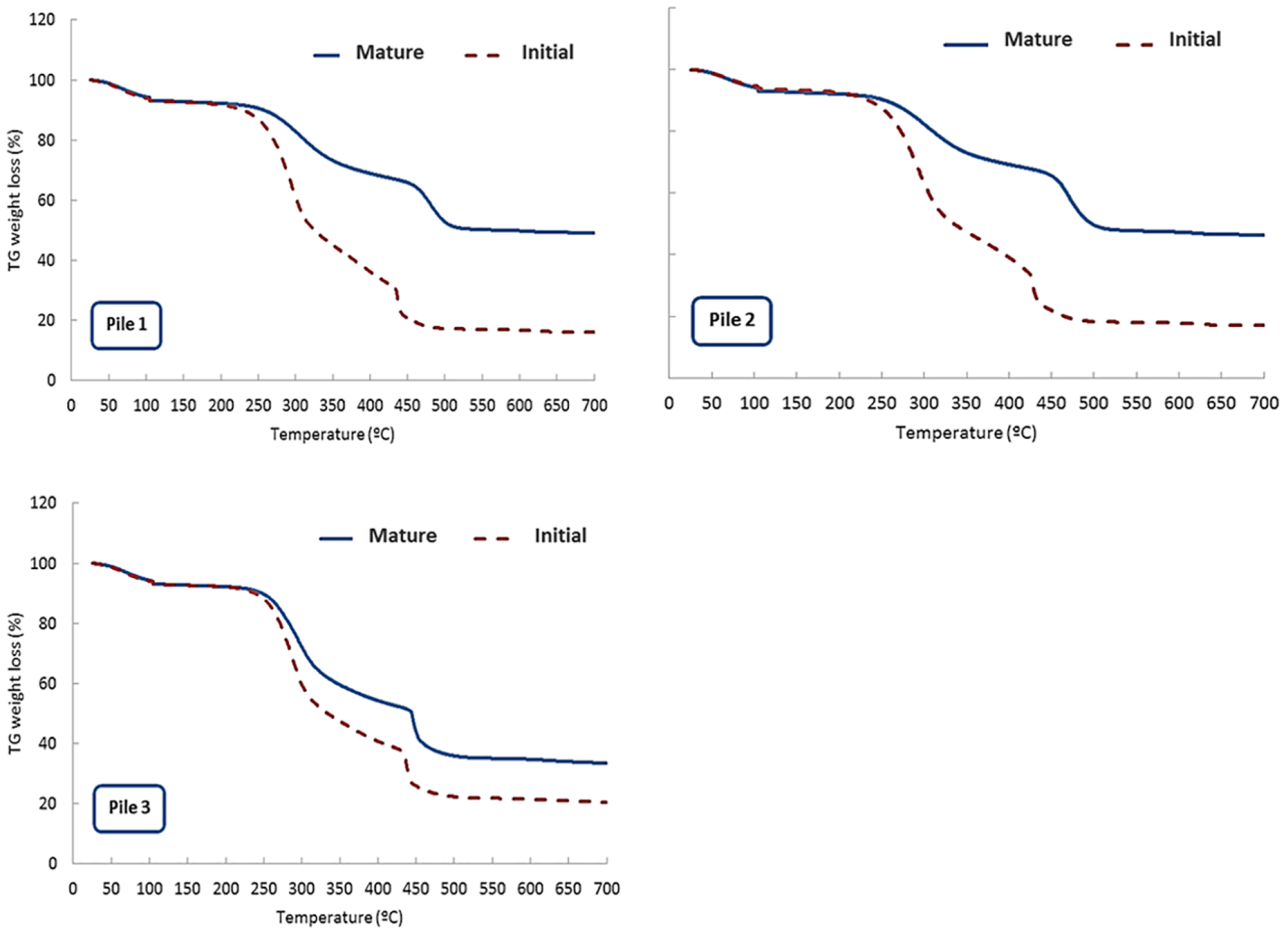
Thermogravimetric (TG) curves for compost samples at the beginning and end of the composting process. P1: 50% MW + 33% TP + 17% PP; P2: 60% MW + 30% TP + 10% PP; P3: 75% MW + 25% PP. MW: food market waste; TP: tree prunings; PP: palm prunings.

The thermograms showed three main steps regions, defined by the different mass losses (Fig 3). The first step is related to the loss of residual water in the 50–150°C range. The second step, corresponding to the highest loss of mass, appeared in the range of 250 to 350°C, this decrease being considerably higher in the samples corresponding to the beginning of the process than for the mature samples. This fact is due to the decomposition of the heat-labile material in the early stages of composting, which produces an accumulation with time of the most recalcitrant material in the composting piles, as it was also reported by Torres-Climent et al. [28] in samples obtained during composting of winery-distillery wastes. Finally, the third step corresponds to the range of 450–500°C, this decrease being more notable in the mature composting samples. The range between 350–500°C has been attributed in different studies to the degradation of complex aromatic structures, such as the humified organic matter [26, 28, 32]. Therefore, the more stabilized is the sample, the more energy takes for decomposition, e.g. to reach the same mass losses requires higher temperature values, due to the greater contents in highly complex aromatic compounds, indicative of the OM stabilization during the composting process [28].

The R1 index increased during the composting process in all the piles (Table 2). This increment in recalcitrance was especially significant in the piles created using TP. However, the presence of PP in the piles seemed to induce the lower changes in this parameter during the process. In this sense, Gregorich et al. [33] reported that temperature at which half of the C was pyrolysed was strongly correlated with mineralizable C. However, Bernal et al. [34] reported that cellulose, hemicellulose and lignin derived components are only partially decomposed during composting and transformed also in a lower speed. Cáceres et al. [35] observed higher temperatures and longer bioactive period in piles with increased vegetable waste. During the composting of horticultural plant wastes, Jurado et al. [25] observed that the fraction most affected by the composting process was cellulose, in contrast to lignin and hemicellulose, whose concentration in the final product remained almost unchanged in comparison to the content in raw material.

**Table 2:** Evolution of compost quality parameters (dry matter basis).

Type of waste	Sampling	P1	P2	P3
pH	Beginning	6.35	6.50	6.80
	End	8.82	8.85	7.43
EC (mS cm^-1^)	Beginning	2.55	2.29	4.40
	End	1.42	1.79	2.02
WSPol (mg kg^-1^)	Beginning	3757	3653	1508
	End	616	616	866
Corg/Nt	Beginning	25.4	23.9	41.7
	End	16.2	11.9	17.5
CEC (meq 100g^-1^ OM)	Beginning	59	53	57
	End	149	155	137
GI (%)	Beginning	0	0	0
	End	81.1	85.3	95.8
R1 index	Beginning	0.25	0.26	0.32
	End	0.73	0.85	0.44

EC: electrical conductivity; WSPol: water-soluble polyphenols; Corg: total organic carbon; Nt: total nitrogen; GI: germination index; CEC: cation exchange capacity; OM: organic matter. P1: 50% MW + 33% TP + 17% PP; P2: 60% MW + 30% TP + 10% PP; P3: 75% MW + 25% PP. MW: food market waste; TP: tree prunings; PP: palm prunings.

Regarding the evolution of the compost quality parameters during the process, pH values increased in all the mixtures during composting, obtaining only values close to neutrality in P3. The significant increase in the pH have been commonly found during composting of vegetable wastes [26, 27, 36], mainly due to the decomposition of acid-type compounds, such as carboxylic and phenolic groups (benzoic, *p*-OH-benzoic, vanillic, veratric, syringic and *p*-coumaric acids [37], and the mineralization of proteins, peptides and amino acids to ammonia [22, 30].

In relation to the salinity, the EC decreased in all the composting piles, showing lower values at the end of the process (Table 2). EC usually tends to increase during composting, due to OM degradation processes, which generate the production of inorganic compounds and the increasing relative concentration of ions due to the mass loss of the pile [38].

However, in this study, the composting process was developed in an open composting facility, which favours salt leaching by watering and especially, by rain. Other composting studies have reported this decrease in salinity due to salt leaching as a consequence of the rain, such as Gavilanes-Terán et al.[27], during composting of horticultural wastes with animal manures and González-Fernández et al. [39] during the co-composting of the waste generated in the guacamole production with garden pruning waste. Therefore, this fact implies the obtaining of composts with salinity contents considerably lower than those obtained in composts elaborated with similar raw materials, but different conditions that do not favour salt leaching [40, 41]. Salinity is one of the main constraints for the use of compost in agriculture, especially as substrate in soilless crop production, since salinity represents the main limiting factor for seed germination and seedling growth, due to the potential phytotoxicity associated to the salt contents [42]. Thus, composting in open facilities constitutes a low-cost procedure, more suitable in developing countries, which has the additional benefit of increasing the quality of the end-products obtained. P1, P2 and P3 showed a reduction of the electrical conductivity of 42%, 22% and 54% compared to the initial values. As an example, Wang et al. [43] reported in a general survey of 104 commercial organic fertilizers from full-scale compost factories in the Jiangsu province (China) a mean value of 5.61 dS/m, a value considerably higher to those obtained in the organic fertilizers obtained in this study.

The concentrations of the water-soluble polyphenols, a specific type of antioxidant phytochemicals naturally present in practically all plant materials [44], strongly decreased throughout the composting process, reaching values lower than 1000 mg kg^-1^. The initial highest values were observed in P1 and P2, the composting piles elaborated with TP, which had the greatest contents in these compounds (Table 1). High levels of these compounds in the compost can produce an adverse environmental impact mainly due to their phytotoxic effect, inhibiting plant germination [45], as well as their effect on soil nitrogen immobilisation [46]. The decrease of the water-soluble polyphenols has been observed in other experiments of composting using raw materials with high contents of these compounds, such as the winery-distillery wastes [22, 28] and the wastes from olive oil production [45].

The germination index in all the final composts showed values greater than 50%, the limit value established by Zucconi et al. [47], which indicates absence of phytotoxins. El Fels et al [48] also reported the efficiency of composting in reducing phytotoxicity, especially in complex composting systems such as sewage sludge-lignocellulosic waste, reaching in the composts obtained values of the germination index greater than 90%, starting from very low values (16–58%).

The cation exchange capacity (CEC) is another parameter strongly associated to the humification process that is usually used as indicator of these processes during composting [34]. This parameter shows the evolution of the humification due to the formation of carboxyl and/or hydroxyphenolic functional groups [22, 49]. In all the composting mixtures, the values of the CEC increased during the process. At the end of the maturation stage, all the compost showed values of CEC higher than that established by Iglesias-Jiménez and Pérez-García [49] (CEC > 67 meq 100 g^-1^), which indicates a suitable degree of maturity in the composts obtained. The Corg/Nt ratio is also considered as an indicator of compost maturity and of the good development of the composting process [34]. At the beginning of the process, all the piles showed values close to 25, except for P3, elaborated only using MW and PP. This parameter decreased considerably in all the mixtures, reaching values in the final composts lower than 20, the value suggested by different authors as indicative of compost maturity [34]. Due to heterogeneity of the raw materials used in the composting processes, which can favour values that fulfil the criterion of the C/N ratio at the beginning of the process, another parameter that considers the initial and final values of this ratio, the T value (T value = the final C/N ratio/the initial C/N ratio) is increasingly used to evaluate organic matter stabilization in composting, considering T values < 0.70 as indicative of compost maturity [18]. In this experiment, T values were 0.64>0.50>0.42 for P1, P2 and P3, respectively, also showing the maturity of the composts obtained.

### Compost as added value organic fertilizer: Quality requirements and economic value

Compost quality and in general, its value as organic fertilizer is still only determined by several standardized values (organic matter, heavy metals, pathogens and nutrients) established in the different national and international legislations and/or guidelines.

Regarding organic matter contents, all the composts practically fulfilled the requirements established in the different guidelines (Table 3). In relation to the concentrations in macroelements, only the guidelines established in USA for compost quality and the ecological criteria for soil improvers [50, 51, 52] considered reference values for these elements, specifically for N and P. Concerning the N contents, all the composts showed values in the range established in these guidelines [50, 51, 52]. However, the concentrations of P were lower in all the composts compared to value considered by the guidelines established in USA [50, 51]. The low contents in P is characteristic in compost elaborated using green wastes, the values obtained being similar to those obtained in composts obtained from vegetable wastes and manures [27, 40, 53].

**Table 3:** Main chemical characteristics, nutrient contents and economic value of the composts obtained (chemical parameters expressed on a dry weight basis).

Parameter	Compost P1	Compost P2	Compost P3	USA guidelines^1^	EU guidelines^2^	EU ecological criteria for soil improvers^3^
OM (%)	53.8	49.6	59.8	*50–60*	*> 15*	*> 20*
*Macroelements (g/kg)*						
Nt	17.9	22.2	18.5	*≥ 10*	*—*	*< 30*
P_2_O_5_	5.7	7.8	4.7	*≥ 10*	*—*	*—*
K_2_O	14.6	20.3	13.6	*—*	*—*	*—*
*Microelements (mg/kg)*						
Fe	2850	2912	13850	*—*	*—*	*—*
Cu	23	26	95	*1500*	*200*	*100*
Mn	83	106	348	*—*	*—*	*—*
Zn	54	62	230	*2800*	*600*	*300*
*Heavy metals (mg/kg)*						
Cd	<0.5	<0.5	1.5	*39*	*1*.*5*	*1*
Cr	30	34	88	*1200*	*100*	*100*
Ni	35	41	108	*420*	*50*	*50*
Hg	<0.5	<0.5	0.8	*17*	*1*	*1*
Pb	15	18	58	*300*	*120*	*100*
*Estimated economic value (€/ton*^a^*)*						
N	7.1	8.8	7.3			
P_2_O_5_	2.3	3.2	1.9			
K_2_O	24.8	34.5	23.1			
Total	34.2	46.5	32.4			

^1^According to the USA guidelines [50, 51]

^2,3^ According to the EC guidelines [52]

^a^ Considering a dry matter content of 75% in commercial compost. The estimated compost price in Ecuador can be established in 40 €/m^3^ [54]. OM: organic matter; Nt: total nitrogen.

In addition, the concentrations of K were similar to those obtained in composts from the agri-food industry [40] and in composts from horticultural wastes from Ecuador [27]. The concentrations of microelements and heavy metals also fulfilled the different guidelines considered, the heavy metals contents being lower than the limits established in the European and American guidelines, except for Cd in the compost from P3 according to the Ecological Criteria for Soil Improvers [52].

To assess the economic value associated to the compost nutrients, the average prices for urea, DAP, and potassium chloride in 2015 were considered according to the World Bank. These prices corresponded in euros/tonne to 241.83; 459.17 and 272.21 for urea, DAP, and potassium chloride respectively. Therefore, the value of the fertilizing units of N, P_2_O_5_ and K_2_O could be averaged in 0.53, 2.27 and 0.55 €/100 kg. In addition, the economic value of the nutrients present in the composts has been calculated assigning a content of 75% of dry matter in all the composts. This demonstrates that the additional value of compost implies a value when its nutrient contents are considered, especially for the potassium value, representing around 70–75% of the total value of the evaluated macronutrients, involving cost savings.

## Conclusions

The proposed strategies for the management and recycling of food market waste streams by co-composting with gardening pruning wastes have shown their feasibility in terms of organic matter mineralization and humification, obtaining composts with suitable characteristics for their agricultural use. All the composting mixtures showed a suitable development of the composting process, with a significant thermophilic phase, completed in a shorter period of time in pile 3. The composts obtained showed absence of phytotoxicity and suitable agronomic properties for their use as organic fertilizers. Therefore, considering all the environmental and economic issues related to waste management (especially the reduction of landfill disposal and the stabilization of highly biodegradable organic streams by low-cost techniques such as composting), it can be concluded that the proposed alternative should be scaled-up in developing areas to reduce and diversify the urban waste streams, producing high quality and balanced organic fertilizers, with a significant economic value in nutrients that could be internalized in the final compost price.

## Supporting information

S1 File Data corresponding to TG analyses. Include data and figures corresponding to TG analyses of each composting pile. P1: 50% MW + 33% TP + 17% PP; P2: 60% MW + 30% TP + 10% PP; P3: 75% MW + 25% PP. MW: food market waste; TP: tree prunings; PP: palm prunings.
(XLSX)
